# Electronic Cigarette Vaping Did Not Enhance the Neural Process of Working Memory for Regular Cigarette Smokers

**DOI:** 10.3389/fnhum.2022.817538

**Published:** 2022-02-18

**Authors:** Dong-Youl Kim, Yujin Jang, Da-Woon Heo, Sungman Jo, Hyun-Chul Kim, Jong-Hwan Lee

**Affiliations:** ^1^Department of Brain and Cognitive Engineering, Korea University, Seoul, South Korea; ^2^Fralin Biomedical Research Institute at VTC, Virginia Tech, Roanoke, VA, United States; ^3^Department of Psychology, Korea University, Seoul, South Korea; ^4^Department of Artificial Intelligence, Kyungpook National University, Daegu, South Korea

**Keywords:** abstinence, electronic cigarette, tobacco cigarettes, functional magnetic resonance imaging, satiety, working memory

## Abstract

**Background:**

Electronic cigarettes (e-cigs) as substitute devices for regular tobacco cigarettes (r-cigs) have been increasing in recent times. We investigated neuronal substrates of vaping e-cigs and smoking r-cigs from r-cig smokers.

**Methods:**

Twenty-two r-cig smokers made two visits following overnight smoking cessation. Functional magnetic resonance imaging (fMRI) data were acquired while participants watched smoking images. Participants were then allowed to smoke either an e-cig or r-cig until satiated and fMRI data were acquired. Their craving levels and performance on the Montreal Imaging Stress Task and a 3-back alphabet/digit recognition task were obtained and analyzed using two-way repeated-measures analysis of variance. Regions-of-interest (ROIs) were identified by comparing the abstained and satiated conditions. Neuronal activation within ROIs was regressed on the craving and behavioral data separately.

**Results:**

Craving was more substantially reduced by smoking r-cigs than by vaping e-cigs. The response time (RT) for the 3-back task was significantly shorter following smoking r-cigs than following vaping e-cigs (interaction: F (1, 17) = 5.3, *p* = 0.035). Neuronal activations of the right vermis (*r* = 0.43, *p* = 0.037, CI = [-0.05, 0.74]), right caudate (*r* = 0.51, *p* = 0.015, CI = [0.05, 0.79]), and right superior frontal gyrus (*r* = −0.70, *p* = 0.001, CI = [−0.88, −0.34]) were significantly correlated with the RT for the 3-back task only for smoking r-cigs.

**Conclusion:**

Our findings suggest that insufficient satiety from vaping e-cigs for r-cigs smokers may be insignificant effect on working memory function.

## Introduction

Cigarette smoking is one of the leading causes of disease, a variety of cancers, and other illnesses. The cigarette consumption level affects the rate of nicotine metabolism (Benowitz et al., 2003) and plasma cotinine is used to indicate the daily intake of nicotine from tobacco cigarettes (Benowitz and Jacob III, 1994). The nicotine influences on body metabolism associated with the reward system (Dileone et al., 2012; Zoli and Picciotto, 2012). In addition, smoking cigarettes is associated with change in body mass index (Munafò et al., 2009) and socioeconomic status (Hiscock et al., 2012) but not the personality (Munafo et al., 2007). Accordingly, a number of reports have been made that the smokers attempted to stop smoking using a wide range of strategies (Bierut, 2009; Fagerström et al., 2012; Brown et al., 2014; Kasza et al., 2014; Mishina and Hoffman, 2014).

The use of the electronic cigarettes (e-cigs), a tobacco-free supplemental nicotine device, has recently become a widespread method of smoking cessation (Eastwood et al., 2015; Ramo et al., 2015; Mirbolouk et al., 2018). Mirbolouk et al. (2018) reported that 4.5% (*n* = 15,240) of 466,842 participants had used an e-cig, which corresponds to 10.8 million adult e-cigarette users in the United States (Mirbolouk et al., 2018). Many behavioral and sociological studies have reported e-cig use on the urge to smoke (Bullen et al., 2010; Dawkins et al., 2013; Kim and Selya, 2020). For example, e-cig use may significantly reduce the desire to smoke, but to a lesser extent than regular tobacco cigarettes (r-cigs) (Bullen et al., 2010). In another study, current e-cig use does not seem to increase the risk of current r-cig smoking, but instead increases the risk of lifetime cigarette smoking (Kim and Selya, 2020).

Despite the growing popularity of e-cigs, only a few studies have reported the neuronal substrates of e-cigs vaping by comparing abstained (ABS) and satiated (SAT) states (Hobkirk et al., 2018), and by analyzing cue reactivity under a naturalistic e-cig smoking condition (Wall et al., 2017) using data taken from functional magnetic resonance imaging (fMRI).

In contrast, a number of fMRI studies have been conducted to reveal the neural substrates underlying r-cig use. These have reported activations in the insula (Naqvi et al., 2007; Benowitz, 2010), limbic areas including the orbitofrontal cortex and amygdala (Stein et al., 1998; Franklin et al., 2007), and the default-mode network (DMN), including the medial prefrontal cortex, posterior cingulate cortices, and angular/supramarginal gyri (Suñer-Soler et al., 2012; Morales et al., 2014; Fedota and Stein, 2015; Janes et al., 2020).

Based on the neuronal substrates identified for cigarette smoking, changes to cognitive functions due to nicotine deprivation have also been widely investigated, particularly concerning working memory load (Ernst et al., 2001b; Jacobsen et al., 2007; Loughead et al., 2015; Nardone et al., 2020). For example, deprived smokers have exhibited lower working memory performance in accuracy and response time (RT) in a 2-back letter recognition task (Nardone et al., 2020).

However, to the best of our knowledge, no previous fMRI studies have investigated the neuronal underpinnings of vaping e-cigs compared to smoking r-cigs. Moreover, no fMRI research has compared the association of cognitive functions with the neuronal substrates of vaping e-cigs and smoking r-cigs. Thus, we were motivated to address these research questions using fMRI data measured for abstained and satiated states in smoking r-cigs and vaping e-cigs.

## Materials and Methods

### Participants

The Institutional Review Board (IRB) at Korea University approved the overall study protocol. All subjects signed a written consent form before the experiment and were compensated according to the IRB document once they had completed the experiment. Twenty-two right-handed healthy young adult males were recruited from a telephone interview from July 2015 to January 2016. Male participants were recruited to eliminate potential confounding factors in neuronal activations due to hormonal differences (Christensen et al., 1984; Michnovicz et al., 1986) and in the sex difference of nicotine metabolism (Benowitz et al., 2003; Munafò et al., 2009; Zoli and Picciotto, 2012).

The inclusion criteria were ≥five years of r-cigs smoking (Shiffman and Paty, 2006; Boulos et al., 2009), ≥ 15 cigarettes per day (Cornelius et al., 2001; Heffner et al., 2013; Selby et al., 2013), a Fagerström Test of Nicotine Dependence (FTND) score of ≥ 4, an Edinburgh Handedness Inventory (EHI) laterality quotient of >75, and the self-reported absence of neurological and/or neuropsychiatric conditions. The majority of the participants were heavy r-cig smokers but were naïve to e-cigs, except for six participants who had experience vaping e-cigs prior to the experiment (only once for two subjects; less than 10 times for one subject; every day for one month for two subjects; and three months for one subject). There was no participant with any substance use disorders based on their verbal report.

During an in-person interview, the participants who met the inclusion criteria responded to a series of questionnaires, including the Perceived Stress Scale, the Beck Depression Inventory, and the Beck Anxiety Inventory. They were also asked to complete the Montreal Imaging Stress Task (MIST) and a 3-back alphabet/digit recognition task to measure their baseline levels for mood and cognitive performance. The MIST consists of a series of computerized mental arithmetic challenges with feedback that consists of a *hit*, *miss*, or *timeout*; it has been effectively used in past research to determine physiological and associated neuronal changes because of the release of cortisol, which is associated with stress perception and processing (Dedovic et al., 2005). A 3-back task was employed to evaluate working memory function. A wide-ranging achievement test for reading was also conducted to evaluate the level of English proficiency required for the MIST.

**FIGURE 1:**
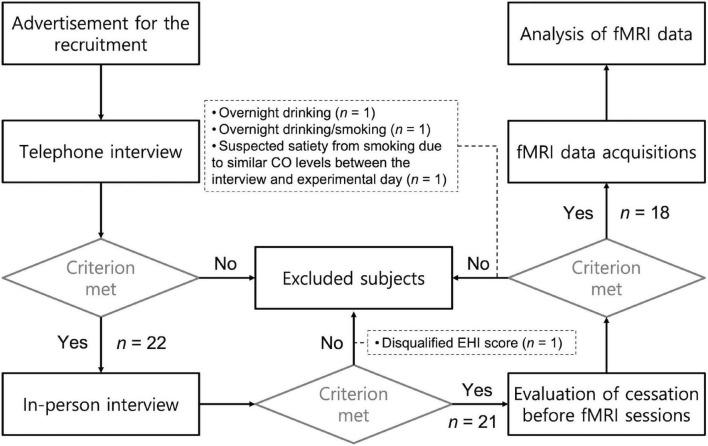
Chematic flowchart for process of subjects screening.

Each of the subjects participated in two sessions that took place on two separate days, one week apart. Participants were asked to refrain from smoking cigarettes or drinking alcoholic beverages overnight before the experimental days. When the participants arried at the MRI center, their craving and carbon monoxide (CO) levels from their exhaled breath were measured. Data from four participants were excluded from the analysis due to (a) overnight drinking before the fMRI session (*n* = 1), (b) overnight drinking and smoking (*n* = 1), (c) a disqualifying EHI score (*n* = 1; Laterality Quotient = 35.5), and (d) suspected satiation from r-cigs smoking due to similar CO levels between the interview and experiment day (less than 10 ppm difference; *n* = 1). The CO level cutoff to determine smoking abstinence was based on the difference between measurements obtained on the interview and experimental days. Figure 1 illustrates the screening procedure for recruiting participants. Table 1 summarizes the demographic and behavioral data obtained from the included participants (*n* = 18).

**TABLE 1:** Demographics and behavioral data for the participants (*n* = 18) obtained on the interview day.

Age (years)	24.9 ± 2.0
Handedness (Laterality Quotient)^1^	86.9 ± 8.6 (α = 0.79 ± 0.13)^2^
CO level (ppm) on the interview day^3^	23.3 ± 4.2
FTND^4^	5.2 ± 1.3
Cigarettes per day	18.0 ± 2.8
Years of cigarette smoking	7.0 ± 1.6
Pack years	8.9 ± 2.6
Beck Depression Inventory (BDI; 0–63)^5^	3.2 ± 2.4
Beck Anxiety Inventory (BAI; 0–63)^6^	3.3 ± 2.5
Perceived Stress Scale (PSS; 0–40)^7^	13.7 ± 5.9
WRAT score for verbal intelligence^8^	89.9 ± 3.5
MIST accuracy (%) on the interview day^9^	58.9 ± 17.2
3-back task accuracy (%) on the interview day	89.4 ± 6.9

*^1^Edinburgh Handedness Inventory test;*

*^2^Mean and STD of Cronbach’s alpha across ten items;*

*^3^CO levels were measured using the piCO Smokerlyzer (Bedfont Scientific Ltd., Rochester, United Kingdom);*

*^4^Fagerström Test of Nicotine Dependence score;*

*^5^minimal depression [0–9], mild depression [10–18], moderate depression [19–29], severe depression [30-63];*

*^6^minimal anxiety [0–7], mild anxiety [8-15], moderate anxiety [16-25], severe anxiety [26-63];*

*^7^rarely stressed [0-10], occasionally stressed [10–20], often stressed [20-30], severely stressed [30-40];*

*^8^WRAT, Wide Range Achievement Test;*

*^9^MIST, Montreal Imaging Stress Task.*

### Behavioral and Craving Data During an MRI Session

Craving levels were measured using the 10-point subjective craving score (CRS; 1 being the minimum and 10 being the maximum level of cigarette craving), the Questionnaire on Smoking Urges (QSU), the Minnesota Withdrawal Scale (MNWS), and the Shiffman-Jarvik Withdrawal Scale – Short Version (SJWS). Additionally, behavioral data from the MIST and the 3-back task were obtained to evaluate baseline affective and cognitive functions, respectively at this time point (pre-MRI).

### MRI Imaging Parameters

A 3-T MRI scanner and 12-channel head coil (Tim Trio, Siemens, Erlangen, Germany) were used to acquire the MRI data. Anatomical images were obtained during the first visit using a T1-weighted pulse sequence using a magnetization-prepared rapid gradient-echo (time-of-repetition [TR] = 1900 ms; time-of-echo [TE] = 2.52 ms, flip angle [FA] = 9° field-of-view [FoV] = 256 × 256 mm^2^; voxel size = 1 mm × 1 mm × 1 mm; slice thickness = 1 mm; number of sagittal slices = 176). Blood-oxygenation-level-dependent (BOLD) fMRI data were acquired using a gradient-echo echo-planar-imaging (EPI) pulse sequence (TR/TE = 2000/30 ms; FA = 90° FoV = 240 mm × 240 mm; voxel size = 3.75 mm × 3.75 mm × 4 mm; slice thickness = 4 mm; 36 axial slices 30° oblique to the anterior-commissure and posterior-commissure line).

### Experimental Paradigm

#### In an Abstained (ABS) State

Two fMRI runs were acquired in an ABS state after overnight cessation of cigarette smoking (165 EPI volumes per run, including five dummy volumes). The two runs for each condition (i.e., abstained or satiated states) per cigarette type (i.e., r-cigs or e-cigs) were acquired and the resulting parameter estimates of neural substrates were averaged to minimize within-subject variability. In each fMRI run, a block-based task paradigm was employed, and in each of the three types of block, the participants watched a series of images (i) with smoking cues (SMK; i.e., a person holding a cigarette/lighter and smoking a cigarette), (ii) with no smoking cues (NTR; i.e., a person holding eyeglass or a pen in their mouth), or (iii) with randomly scrambled phases of the matched SMK/NTR images as a fixation condition (FIX) that were used to obtain the baseline BOLD intensities in comparison to the higher BOLD intensities from the intact SMK/NTR images (Lee et al., 2012; Kim et al., 2015). Participants were asked to watch the SMK, FIX, or NTR images through MR-compatible visual goggles (NordicNeuroLab)^1^, and confirmed that they saw each image by pressing a button on a fiber optic response pad (Current Design)^2^. They were asked to focus on their craving when the SMK images were shown. Five SMK and NTR blocks were interleaved with FIX blocks in one fMRI run, with five images (3 s per image) in each block (Lee et al., 2012; Kim et al., 2015). Participants who did not press the button for two consecutive images during fMRI scanning were suspected to be drowsy, and thus the corresponding run was excluded from analysis; this occurred for a total of four runs for four participants.

#### In a Satiated (SAT) State

After the two fMRI runs in an ABS state, the participants were removed from the MRI scanner and requested to smoke cigarettes until satiation. The decision of whether to smoke an e-cig or r-cig during the first visit was determined by a coin toss, with the participants smoking the other type of cigarette during the second visit. Under the r-cig smoking condition, the number of cigarettes smoked was counted, while the number of puffs was counted for the e-cig smoking condition. The e-cigs used in our experiment was the Haka S2^3^, and the liquid was Virginia-flavored Magnificent 7^4^, which contains a plasma nicotine concentration of 16 mg/ml. After smoking the e-cigs or r-cigs, CO levels and the scores for the four craving questionnaires (i.e., CRS, QSU, MNWS, and SJWS) were recorded during the after-smoking phase. It took approximately 10 min from the start of cigarette smoking to the start of the first fMRI run in the SAT state. This was long enough for the nicotine to be absorbed into the bloodstream and to reach the brain, thus completely dissipating throughout the nervous system (Stein et al., 1998; Dawkins et al., 2013). Participants were then placed back in the MRI scanner, and two fMRI runs were acquired in the SAT state in the same manner as for the ABS state. After fMRI data acquisition, CO levels and the scores for the four craving questionnaires were recorded; MIST and 3-back task performance was observed during the post-MRI phase.

### Analysis of Physiological and Behavioral/Cognitive Data

The collected physiological and behavioral/cognitive data (i.e., CO levels, CRS, QSU, MNWS, SJWS, MIST, and the 3-back task) for each of the e-cig and r-cig smoking conditions and for the three-time points (i.e., pre-MRI, after smoking, and post-MRI) were analyzed using two-way repeated-measures ANOVA as implemented in the SPSS software toolbox (IBM Corp., 2013; IBM SPSS Statistics for Windows, Version 22.0. Armonk, NY: IBM Corp.). Cronbach’s alpha was measured the consistency across the three-time points for each of the cigarette types and the inconsistency between smoking r-cigs and vaping e-cigs.

### Analysis of fMRI Data

#### Preprocessing

Raw EPI volumes were preprocessed using the Analysis of Functional NeuroImages (AFNI) software toolbox^5^. The preprocessing steps included (1) removing spikes from the BOLD time series using 3dDespike, (2) slice timing correction using 3dTshift, (3) aligning anatomical images and EPI images using align_epi_anat.py, (4) warping anatomical images to the Montreal Neurological Institute template space using @auto_tlrc, (5) volume registration using 3dvolreg, (6) spatial smoothing with 8-mm full-width at half-maximum using 3dmerg, (7) in-brain mask generation using 3dAutomask, (8) detrending using 3dDetrend, and (9) the scaling of BOLD signals to the mean of 100 using 3dcalc.

#### Estimation of Neuronal Activations and Group Inference

Preprocessed BOLD fMRI volume series concatenated across all the eight runs (i.e., two runs in the ABS or SAT conditions for each of the two cigarette types) were analyzed using a general linear model (GLM) to estimate neuronal activations at an individual level by using 3dDeconvolve and 3dREMLfit for generalized least squares in AFNI. The “@ANATICOR” script in AFNI was used to regress out the cardiorespiratory noise, the motion parameters, and their first-order derivatives in the BOLD signal (Jo et al., 2010) to avoid mixing contributions from different tissue types (Jo et al., 2013, 2021). The contrast of the effect sizes (i.e., the beta values from the GLM) between the SMK and NTR blocks were then obtained for each of the SAT and ABS conditions. Consequently, paired *t*-tests were employed to identify the brain regions that showed significantly different neuronal activations between the SAT and ABS conditions at the group level by fixing the cigarette type (i.e., e-cig or r-cig). Potential outliers for the neuronal activations across the subjects were identified based on the median absolute deviation [MAD; (Rangaprakash et al., 2018; Behroozi et al., 2020; Prabhakaran et al., 2021)] and subsequently removed from the voxel-wise group inference (Kim et al., 2019b; Jo et al., 2021). The statistical significance used to identify the regions-of-interest (ROI) was corrected using a null distribution of 10,000 group inference tests obtained from 10,000 sets of randomly permuted labels across the ABS and SAT conditions (Nichols and Holmes, 2002; Kim et al., 2020).

#### Interpretation of Regions-of-Interest Using Regression Analysis

Regions-of-interest identified from the group inference were further interpreted using ROI-wise regression analysis. Simple linear regression analysis was adopted to find association between the difference of neuronal activations (i.e., ABS vs. SAT) for each ROI and the physiological, questionnaire, and/or cognitive data. To evaluate the association of the ROIs with working memory performance, the regression analysis was conducted using either the RT or the accuracy for the 3-back task. Potential outliers for the RT and accuracy were identified based on the MAD (Rangaprakash et al., 2018; Behroozi et al., 2020; Prabhakaran et al., 2021) and subsequently removed during the regression analysis (Kim et al., 2019b). The statistical significance of the resulting correlation coefficients was corrected using random permutations (*n* = 10,000), in which the regression analyses were conducted using randomly shuffled indices for the ABS and SAT conditions to generate a null distribution and consequently to obtain a corrected *p*-value (Perrett et al., 2006; Kim et al., 2015). Also, the 95% confidence interval (CI) for the correlation coefficients was obtained from 10,000 cycles of bootstrapping with replacement (Kim et al., 2019b) which has been widely adopted as non-parametric approach such as in the economy (Briggs et al., 1997), engineering (Saha et al., 2018), medicine (Barber and Thompson, 2000), and statistic (Simar and Wilson, 2000). Regression analysis using the estimated contrast of neuronal activations from the ROIs was similarly conducted for the RT or the accuracy from the MIST or the physiological/questionnaire data related to the craving, followed by the statistical significance correction. The reliability of regression analysis was evaluated using the one-way ANOVA based intra-class correlation coefficient (ICC; (Zuo et al., 2010; Kim and Lee, 2011)).

## Results

### Behavioral Data

The EHI scores across participants showed relatively high internal consistency (Cronbach’s α = 0.79). The hours of overnight abstinence for the e-cig (11.3 ± 2.4 h) and r-cig (11.2 ± 1.3 h) sessions were not statistically different (*p* = 0.68 from a paired *t*-test). Participants smoked 1.6 (± 0.6) r-cigs (*n* = 16 due to missing data from two subjects) and had 12.2 (± 4.1) puffs of the e-cigs (*n* = 17 due to missing data from one subject). Eight subjects vaped an e-cig during the first visit, and the remaining ten subjects smoked an r-cig during the first visit.

### Physiological, Questionnaire, and Cognitive Performance Data

**FIGURE 2:**
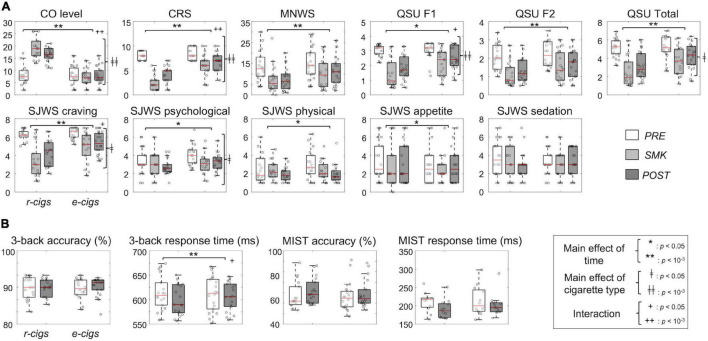
Behavioral data analysis: (A) Physiological data across the three time points on an experimental day (i.e., PRE, pre-MRI; SMK, after smoking; POST, post-MRI) and two cigarette types and (B) accuracy and response time of the cognitive tasks between the two time points (i.e., PRE and POST) and two cigarette types. The *p*-value was Bonferroni-corrected. CO, carbon monoxide; MNWS, Minnesota Withdrawal Scale; QSU, Questionnaire on Smoking Urges; SJWS, Shiffman-Jarvik Withdrawal Scale – Short Version; r-cigs, regular tobacco cigarettes; e-cigs, electronic cigarettes; RT, response time; MIST, Montreal Imaging Stress Task.

Figure 2 presents the CO levels results, subjective craving data from the questionnaires, 3-back task, and MIST acquired for each of the three-time points during each of the two MRI sessions under either the r-cig or e-cig smoking condition (see Supplementary Tables 1, 2 for details). There was a significant main effect of cigarette type for the CO levels, CRS, and craving-related scores acquired from the four questionnaires. For examples, the CRS right after smoking was significantly lower for the r-cig condition than for the e-cig condition (*F*(1, 17) = 45.4, Bonferroni-corrected *p* < 10^–3^, Cronbach’s α = 0.60), while the SJWS scores were also substantially reduced (Bonferroni-corrected *p* < 0.05) for the r-cigs than for the e-cigs in the craving (*F*(1, 17) = 7.0, Cronbach’s α = 0.20) and psychological sections (*F*(1, 17) = 4.5, Cronbach’s α = 0.58). In addition, there was a significant main effect of time for the majority of the craving-related scores except for the SJWS score in the sedation section. A significant interaction effect between the cigarette type and time was also observed for the craving-related measurements such as the CO levels, CRS, QSU-F1/Total, and SJWS craving/psychological sections, possibly due to the substantial reduction in the craving levels for the r-cigs compared to the e-cigs. Interestingly, there was a significant main effect of time (Bonferroni-corrected *p* < 0.001) and of the interaction (Bonferroni-corrected *p* < 0.05) for the RT of the 3-back task. However, there was no significant main effect or interaction for the accuracy of the 3-back task. There was also no main effect or interaction for MIST performance (Bonferroni-corrected *p* > 0.05).

### Brain Regions Associated With the Cigarette Smoking Conditions

**FIGURE 3:**
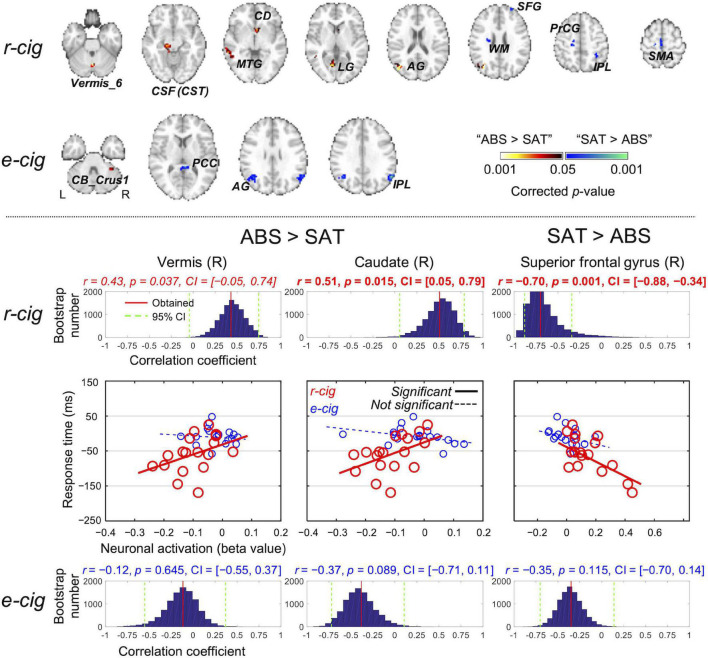
Regions-of-interest (ROIs) identified from the contrast of the satiated and abstained states for each of the two cigarette smoking conditions (top). A significant association between the neuronal activations of the ROIs and the response time for the 3-back alphabet/digit recognition task was obtained using regression analysis only from the three ROIs identified from the r-cig smoking condition (bottom). The corresponding *p*-value was corrected using 10,000 random permutations, and the statistical significance was evaluated based on the 95% confidence interval (CI) obtained from 10,000 bootstrapping cycles. L, left; R, right; SAT, satiated state; ABS, abstained state; CB, cerebellum; PCC, posterior cingulate cortex; AG, angular gyrus; IPL, inferior parietal lobule; CSF, cerebral spinal fluid; CST, corticospinal tract; MTG, middle temporal gyrus; CD, caudate; LG, lingual gyrus; WM, white matter; SFG, superior frontal gyrus; PrCG, precentral gyrus; SMA, supplementary motor area; e-cigs, electronic cigarettes; r-cigs, regular tobacco cigarettes.

Figure 3 shows the ROIs identified from the smoking of each of the two cigarette types (see Supplementary Table 3 for details). Of the ROIs, the lower neuronal activations (i.e., beta values from the GLM) in the SAT state for the right vermis (*r* = 0.43, *p* = 0.037, CI = [-0.05, 0.74], ICC = 0.49) and right caudate (*r* = 0.51, *p* = 0.015, CI = [0.05, 0.79], ICC = 0.36) after r-cig smoking had weakly suggestive and significantly positive correlations with the lower RT for the 3-back task, respectively. On the other hand, the increased neuronal activations of the right superior frontal gyrus (*r* = −0.70, *p* = 0.001, CI = [−0.88, −0.34], ICC = −0.73) in the SAT state for the r-cig smoking only had a significant correlation with the lower RT for the 3-back task. There was no significant association between the neuronal activations of the ROIs and either the accuracy for the 3-back task or the RT/accuracy for the MIST.

## Discussion

We investigated the neuronal substrates of smoking r-cigs and vaping e-cigs by contrasting abstinence and satiety for r-cig smokers. fMRI data were obtained to estimate neuronal activations, while physiological and behavioral data were employed to obtain craving levels, stress response, and working memory performance. Based on our findings, both the physiological and behavioral data related to nicotine craving suggested that r-cig smokers were not fully satiated by vaping e-cigs. The RT for the 3-back task was slower following vaping e-cigs than following smoking r-cigs. In the ROIs identified from the contrast of the SAT versus ABS conditions, the neuronal activations of the right vermis, right caudate, and right superior frontal gyrus exhibited significant associations with the RT for the 3-back task only under the r-cig smoking condition. As far as we are aware, our study is the first to report using fMRI, lower satiety, and compromised working memory performance for vaping e-cigs compared to smoking r-cigs among r-cig smokers.

Several previous studies have reported that smoking deprivation reduced cognitive functions related to working memory (Ernst et al., 2001b; Xu et al., 2005; Mendrek et al., 2006; Jacobsen et al., 2007; Jasinska et al., 2014). More specifically, the RT during working memory task is slower during abstinence than during satiation (Ernst et al., 2001b; Xu et al., 2005; Mendrek et al., 2006). For example, smokers who performed N-back tasks in an ABS state (> 12 h) exhibited a longer RT compared to an SAT state (Xu et al., 2005). Our findings of a lower RT when performing the working memory task in an SAT condition for r-cig smoking is in line with these previous studies. Furthermore, there have been a number of reports that nicotine altered the RT across several cognitive functions (Ernst et al., 2001a; Robles et al., 2008; Fernandes et al., 2019; Scholten et al., 2019; Almeida et al., 2020; Silva et al., 2021a,b). For example, smokers who had excessive use of cigarettes showed a slower RT of Go/No-Go task (Silva et al., 2021a) and visual attention task (Fernandes et al., 2019). Thus, our reported findings of RT can be further cross-validated using previous reports that have been investigated association between the nicotine exposure and the working memory process.

We reported that the right vermis and right caudate showed a significant correlation between neuronal responses and the RT for the 3-back task. This is in line with earlier studies that have reported a slower RT associated with increased neuronal activations in the vermis (Durazzo et al., 2007; Sutherland et al., 2016) and caudate (Lawrence et al., 2002; Kumari et al., 2003; Brody et al., 2006). In the review paper (Jasinska et al., 2014), the effect of acute nicotine administration on cognitive brain functions such as attention and working memory was described, with the working memory circuit including the vermis and caudate along with the frontal areas found to be influenced by nicotine. We also observed that increased neuronal activation in the superior frontal gyrus significantly correlated with the reduced RT for the 3-back task for the r-cig condition only (Ernst et al., 2001b; Lawrence et al., 2002; Xu et al., 2005). Alternatively, ROIs such as the dorsolateral prefrontal cortex and hippocampus in the mesocortical pathway (Zakiniaeiz et al., 2019; Dukes et al., 2020) may also be involved. Thus, the association between the neuronal activations of these candidate ROIs and working memory performance can be investigated.

Determining whether the RT for working memory tasks can be predicted using neuronal activation is an important direction for future research in order to quantify this relationship, which could be achieved by deploying machine-learning and deep-learning-based predictive models (Kim et al., 2019a). Interesting future research could also include real-time fMRI neurofeedback methods (Lee et al., 2009; Kim et al., 2015, 2019b) to investigate whether non-invasive voluntary self-regulation of neuronal activations and/or connectivity can enhance the working memory and otherwise compromised cognitive functions due to nicotine deprivation.

At the debriefing session, all of the participants except one reported that e-cigs vaping did not genuinely feel like smoking a cigarette. This may be because the majority of participants were heavy r-cig smokers. Some used the analogy that “vaping electronic cigarettes was like drinking pure water, whereas smoking regular cigarettes was like drinking a soft drink.” Previous reports have indicated that e-cig vaping may result in a lower degree of craving relief and a stiff burning sensation in the throat caused by the nicotine in the inhaled smoke (Caputi, 2017). Our data suggested that the throat-hit from r-cig smoking was much more vital than that of e-cig vaping, which might also contribute to the lower reduction in craving following the smoking of e-cigs compared to r-cigs. It would be interesting to investigate the relationship between neuronal activations and craving/cognitive performance from heavy e-cig smokers.

The number of brain regions identified by contrasting the SAT and ABS states was higher under the r-cig smoking condition than under the e-cig smoking condition. Several brain regions had a significant association between neuronal activations and craving-related measurements (data not shown here). Under the e-cig smoking condition, the neuronal activations from the posterior cingulate cortex only exhibited a significant relationship with the craving-related measurements. Under the r-cig smoking condition, the left angular gyrus, left middle temporal gyrus, left substantia nigra near the fourth ventricle, left white matter near the cingulate gyrus, and right inferior parietal lobule (Supplementary Table 3) demonstrated a significant association between their neuronal activations and craving levels (Jacobsen et al., 2007; Loughead et al., 2015).

The MIST task assesses the stress response; however, the accuracy and response time were not reported as significantly different across the smoking states and cigarette types. Thus, the accuracy and response time of the MIST task were not included in the *post hoc* regression analysis. Multiple regression analysis using several behavioral data across the 3-back and MIST tasks would further enhance a statistical rigor with a careful consideration of potential multicollinearity issue. The number of participants was relatively low; thus, we have performed the *p*-value correction via random permutations when identifying ROIs and *p*-value correction as well as bootstrapping when analyzing the association between the RT and neuronal activations. Despite our effort to alleviate the weakness of a relatively small sample size, future research recruiting more participants is warranted to evaluate the brain regions associated with the stress response with potentially greater statistical significance than our results (Allenby et al., 2020). In addition, the distribution of our relatively small samples may deviate from population normal distribution; accordingly, we applied random permutation and non-parametric bootstrapping with replacement to resolve the potential bias of statistical inference (Briggs et al., 1997; Barber and Thompson, 2000; Simar and Wilson, 2000; Saha et al., 2018). Alternative methods such as the non-parametric Bayesian models and Spearman’s rank correlation coefficients would be valuable approaches to test the association between the neuronal activations and behavioral data. Also, we recruited only males, so it is not straightforward to generalize our findings to females. The report that the nicotine metabolism that has been associated with a body weight has been discouraging for females who try to quit smoking (Munafò et al., 2009; Zoli and Picciotto, 2012). Thus, the sex-specific effects of nicotine addiction (Christensen et al., 1984; Michnovicz et al., 1986; Zakiniaeiz et al., 2019; Lin et al., 2020) need to be investigated to provide evidence for the development of sex-specific therapy to modulate smoking craving in consideration with nicotine metabolism.

Additionally, distinct psychoactive effects have been reported depending on the types of e-cig device (Keamy-Minor et al., 2019; Holt et al., 2021), the experience of e-cig use (Loud et al., 2021), and types of nicotine products (Ryu et al., 2021). For example, Loud and colleagues have described that varying psychoactive effects, including intensifying the current moods, causing an adrenaline rush, and triggering addiction, are elicited by smoking r-cigs or vaping e-cigs in heavy smokers, dual users of regular and electronic cigarettes, and former smokers who quit smoking (Loud et al., 2021). Some state-dependent influences, such as emotion and motivation, may affect the working memory performance (Blasiman and Was, 2018). Future research is thus warranted to identify an experimental design approach to determining the association between cigarette types and psychoactive effects.

In our study design, the administration parameters such as the number of cigarettes smoked, the nicotine content in r-cigs, and/or the frequency of puffing e-cigs were not controlled. Previous studies have reported the variability of the CO levels and/or nicotine concentrations depending on the frequency of puff (Trtchounian et al., 2010), the type of e-cigs (Omaiye et al., 2021), the number of puff duration (Son et al., 2020), and the duration of smoking cigarettes (Ebajemito et al., 2020). In our study, participants were allowed to smoke r-cigs or to vape e-cigs until their nicotine craving were satiated, and this might have caused the variability in the nicotine exposure. Future research that controlled the administration parameters of nicotine is warranted to investigate the fine-grained information on the satiation from e-cigs compared to r-cigs.

Although most participants in this study were naïve to e-cigs, the degree of liking and/or familiarity with the e-cigs that we prepared would be a confounding factor. In this context, previous research demonstrated that the e-cigs flavor has impacted to reduce cigarette use and quit smoking (Yang et al., 2020); the flavor to e-cigs was highly associated with nicotine consumption (Patten et al., 2021); and the administration of puff inhalation, breath-hold, and exhalation may increase control over amounts of acute exposure to cigarette use (Perkins and Karelitz, 2020). However, Perkins and Karelitz reported that the number of puffs and the duration of puff were different between instructed and uninstructed procedures (Perkins and Karelitz, 2020); it is possible that the smoking craving may not be completely satiated. The participants in our study vaped an e-cig with one fixed flavor. The degree of satiety can be increased if participants vape an e-cig with their preferred flavor, which can be examined during an interview session; therefore, an extension of this study could include control condition of using the preferred flavor of e-cigs. In addition, the familiarity with the product may have an effect on the degree of satiety (Dawkins et al., 2009; Patten et al., 2021) since participants were allowed to smoke their own products for the r-cig condition, in which the familiarity might be an important confounding factor.

There is evidence that the degree of nicotine absorption and the level of nicotine concentration might be different between the use of r-cigs and e-cigs (Farsalinos et al., 2015; O’connell et al., 2019; Yingst et al., 2019). In our study, all the participants completed the physiological and behavioral tests after smoking r-cigs or vaping e-cigs, which took approximately 10 min from the start of r-cigs smoking or e-cigs vaping to the start of the fMRI run in the SAT state. Despite this experimental setup, the absorption of nicotine from e-cigs appears to be slower than that from r-cigs due to the transbuccal absorption for e-cigs (Devito and Krishnan-Sarin, 2018; Yingst et al., 2019). Thus, the fMRI run in the SAT state for the e-cigs vaping condition may have been acquired before the nicotine was sufficiently absorbed. Further research is thus warranted to consider the quantitative measurement of the absorbed nicotine level such as from the blood sample (Farsalinos et al., 2015; Devito and Krishnan-Sarin, 2018; O’connell et al., 2019; Yingst et al., 2019).

## Conclusion

We reported that nicotine cravings were less satiated when vaping e-cigs than when smoking r-cigs for r-cig smoking participants, leading to differences in their neuronal substrates as measured from fMRI data. In particular, the reduced RT for the 3-back alphabet/digit recognition task with SAT compared to ABS under the r-cig smoking condition, but lesser degree under the e-cig vaping condition, is worth noting. The reduced RT had a significant correlation with the neuronal activations in the right vermis, right caudate, and right superior frontal gyrus. We would suggest that our method and findings are informative for future functional neuroimaging studies that seek to investigate potential differences in cognitive and/or affective functions with e-cig and/or r-cig smoking.

## Data Availability Statement

The raw data supporting the conclusions of this article will be made available by the authors, without undue reservation.

## Ethics Statement

The studies involving human participants were reviewed and approved by The Institutional Review Board (IRB) at Korea University. The patients/participants provided their written informed consent to participate in this study.

## Author Contributions

D-YK, D-WH, SJ, and J-HL were responsible for the study concept and design. D-YK, D-WH, SJ, H-CK, and J-HL contributed to the acquisition of data. D-YK, YJ, D-WH, and J-HL assisted with data analysis and interpretation of findings. D-YK and J-HL drafted the manuscript. D-YK, D-WH, H-CK, and J-HL provided critical revision of the manuscript for important intellectual content. All authors critically reviewed content and approved the final version for publication.

## Conflict of Interest

The authors declare that the research was conducted in the absence of any commercial or financial relationships that could be construed as a potential conflict of interest.

## Publisher’s Note

All claims expressed in this article are solely those of the authors and do not necessarily represent those of their affiliated organizations, or those of the publisher, the editors and the reviewers. Any product that may be evaluated in this article, or claim that may be made by its manufacturer, is not guaranteed or endorsed by the publisher.

## Footnotes

1
www.nordicneurolab.com

2
www.curdes.com

3
haka-america.com

4
www.diyflavorshack.com

5
https://afni.nimh.nih.gov
