# Supplementary material for: Electronic Cigarette Vaping Did Not Enhance the Neural Process of Working Memory for Regular Cigarette Smokers
Source: Front Hum Neurosci. 2022 Feb 18;16:817538. doi: 10.3389/fnhum.2022.817538 (PMC8894252; doi:10.3389/fnhum.2022.817538)
Supplement: Supplementary file 1 [file Data_Sheet_1.pdf]

Supplementary Materials

Table S1. Physiological data and behavioral data related to nicotine craving acquired before the MRI scan, after smoking the cigarettes, and after the MRI scan from each of the two visits. Data were analyzed using a two-way repeated measures analysis-of-variance (ANOVA) test and resulting *p*-values were Bonferroni-corrected (significant results were bold-faced).

		smoking condition	*Pre-MRI*	*After smoking*	*Post-MRI*	*C α*	*Main effect of cigarette*	*Main effect of time*	*Interaction*
	CO level	r-cigs	8.1 ± 3.7	19.8 ± 5.7	17.3 ± 4.9	0.76	**F(1, 17) = 52.5<br>*p* < 0.001**	**F(2, 16) = 117.0<br>*p* < 0.001**	**F(2, 16) = 100.4<br>*p* < 0.001**
		e-cigs	8.0 ± 3.7	7.6 ± 3.3	7.8 ± 3.7	0.98			
	CRS	r-cigs	8.0 ± 0.8	2.5 ± 1.6	4.3 ± 1.8	0.55	**F(1, 17) = 45.4<br>*p* < 0.001**	**F(2, 16) = 56.5<br>*p* < 0.001**	**F(2, 16) =24.5<br>*p* < 0.001**
		e-cigs	8.3 ± 1.2	6.1 ± 2.1	6.8 ± 1.8	0.65			
QSU	*Factor1*	r-cigs	6.1 ± 0.7	2.8 ± 2.0	3.7 ± 1.7	0.76	**F(1, 17) = 6.1<br>*p* = 0.024**	**F(2, 16) = 27.6<br>*p* < 0.001**	**F(2, 16) = 5.3<br>*p* = 0.017**
		e-cigs	6.0 ± 1.3	4.5 ± 1.8	5.1 ± 1.6	0.64			
	Factor2	r-cigs	4.2 ± 1.4	2.2 ± 1.3	2.6 ± 1.3	0.81	F(1, 17) = 2.5<br>*p* = 0.133	**F(2, 16) = 21.5<br>*p* < 0.001**	F(2, 16) = 0.8<br>*p* = 0.475
		e-cigs	4.4 ± 1.4	3.0 ± 1.6	3.3 ± 1.5	0.82			
	Total	r-cigs	5.1 ± 1.0	2.5 ± 1.6	3.1 ± 1.5	0.79	**F(1, 17) = 4.5<br>*p* = 0.048**	**F(2, 16) = 26.6<br>*p* < 0.001**	F(2, 16) = 3.3<br>*p* = 0.063
		e-cigs	5.2 ± 1.2	3.7 ± 1.5	4.2 ± 1.4	0.68			
	MNWS	r-cigs	13.7 ± 7.3	7.2 ± 6.4	7.7 ± 5.5	0.83	F(1, 17) = 3.2<br>*p* = 0.094	**F(2, 16) = 10.6<br>*p* < 0.001**	F(2, 16) = 1.3<br>*p* = 0.304
		e-cigs	14.9 ± 7.6	10.1 ± 7.2	11.3 ± 6.8	0.89			
SJWS	*Appetite*	r-cigs	3.3 ± 2.0	2.6 ± 2.1	3.1 ± 2.2	0.92	F(1, 17) = 0.3<br>*p* = 0.620	**F(2, 16) = 6.3<br>*p* = 0.010**	F(2, 16) = 0.3<br>*p* = 0.783
		e-cigs	2.8 ± 1.7	2.5 ± 1.5	2.9 ± 1.8	0.84			
	Craving	r-cigs	6.2 ± 0.6	3.4 ± 1.8	4.3 ± 1.4	0.68	**F(1, 17) = 7.0<br>*p* = 0.017**	**F(2, 16) = 33.3<br>*p* < 0.001**	**F(2, 16) = 3.8<br>*p* = 0.045**
		e-cigs	6.4 ± 0.6	4.9 ± 1.6	5.3 ± 1.2	0.69			
	Physical	r-cigs	2.5 ± 1.5	2.4 ± 1.0	2.0 ± 0.8	0.77	F(1, 17) = 1.1<br>*p* = 0.313	**F(2, 16) = 4.7<br>*p* = 0.025**	F(2, 16) = 0.7<br>*p* = 0.533
		e-cigs	3.0 ± 1.6	2.3 ± 1.1	2.0 ± 1.0	0.67			
	Psycho logical	r-cigs	3.7 ± 1.1	2.7 ± 1.1	2.7 ± 0.9	0.75	**F(1, 17) = 4.5<br>*p* = 0.049**	**F(2, 16) = 8.4<br>*p* = 0.003**	F(2, 16) = 1.5<br>*p* = 0.258
		e-cigs	4.0 ± 1.1	3.1 ± 1.0	3.3 ± 0.9	0.58			
	Sedation	r-cigs	3.8 ± 1.8	3.2 ± 1.7	3.1 ± 1.5	0.76	F(1, 17) = 0.1<br>*p* = 0.744	F(2, 16) = 1.3<br>*p* = 0.292	F(2, 16) = 1.3<br>*p* = 0.297
		e-cigs	3.3 ± 1.5	3.1 ± 1.3	3.4 ± 1.4	0.87			

Cig, cigarette; C *α*, Cronbach's alpha; r-cigs, regular tobacco cigarettes; e-cigs, electronic cigarettes; CO, carbon monoxide; CRS, craving score (0-10); QSU, Questionnaire on Smoking Urges (factor 1: strong craving and intention to smoke, factor 2: anticipation of negative affect changes after smoking); MNWS, Minnesota Withdrawal Scale; SJWS, Shiffman-Jarvik Withdrawal Scale; n/a, not available.

Table S2. The 3-back alphabet/digit recognition task and MIST performance acquired before and after the MRI scan at each of the two visits. Data were analyzed using a two-way repeated measures ANOVA test and resulting *p*-values were Bonferroni-corrected (significant results were bold-faced).

		Cig. type	*Pre-MRI*	*Post-MRI*	*C α*	*Main effect of cigarette*	*Main effect of time*	*Interaction*
MIST	*Accuracy*	r-cigs	51.3 ± 5.1	54.2 ± 5.2	0.92	F(1, 11) = 1.0<br>*p* = 0.334	F(1, 11) = 2.3<br>*p* = 0.159	F(1, 11) = 0.3<br>*p* = 0.621
		e-cigs	50.4 ± 5.3	52.0 ± 3.6	0.87			
	Response time	r-cigs	209.0 ± 27.3	193.2 ± 24.7	0.74	F(1, 11) = 0.6<br>*p* = 0.446	F(1, 11) = 3.8<br>*p* = 0.078	F(1, 11) = 0.1<br>*p* = 0.797
		e-cigs	213.9 ± 39.2	201.4 ± 30.8	0.75			
3-back task	*Accuracy*	r-cigs	93.6 ± 7.3	94.3 ± 3.9	0.93	F(1, 17) = 0.6<br>*p* = 0.454	F(1, 17) = 2.5<br>*p* = 0.132	F(1, 17) = 2.5<br>*p* = 0.130
		e-cigs	93.2 ± 7.6	93.9 ± 5.8	0.85			
	Response time	r-cigs	654.9 ± 135.9	596.3 ± 123.7	0.42	F(1, 17) = 0.3<br>*p* = 0.572	**F(1, 17) = 29.2**<br>***p* < 0.001**	**F(1, 17) = 5.3**<br>***p* = 0.035**
		e-cigs	641.9 ± 140.4	627.5 ± 116.6	0.93			

Cig, cigarette; C α, Cronbach's alpha; r-cigs, regular tobacco cigarettes; e-cigs, electronic cigarettes; MIST, Montreal Imaging Stress Task.

Table S3. Brain regions identified from a paired *t*-test across the abstinent (ABS) and satiety (SAT) conditions by fixing a cigarette type (also visualized in top row of Fig. 2). Resulting *p*-values were corrected using 10,000 random permutations and voxel-clusters with a minimum of 20 contiguous voxels survived from corrected $p < 0.05$ were reported.

Cluster index	***Brain region***	***Side***	***MNI coordinate* (x, y, z in mm)**	***Number of voxels***	***Peak t-score***	***Corrected p-value***
			Electronic cigarettes			
			ABS > SAT			
1	Cerebellum_crus1	R	33, −45, −39	20	4.44	0.048
			SAT > ABS			
1	Posterior cingulate cortex	L/R	3, −39, 12	22	5.38	0.038
2	Angular gyrus	L	−42, −60, 36	76	5.03	0.041
3	Inferior parietal lobule	R	57, −60, 36	74	5.65	0.032
			Regular tobacco cigarettes			
			ABS > SAT			
1	Vermis_6	L/R	0, −69, −27	24	5.80	0.029
2	Fourth ventricle near the substantia nigra	L	−18, −24, −9	27	4.97	0.043
3	Middle temporal gyrus	L	−57, −45, 3	50	5.93	0.025
4	Caudate	R	3, 12, 3	20	5.11	0.040
5	Lingual gyrus	L	−9, −69, 9	32	5.55	0.033
6	Angular gyrus	L	−45, −75, 24	56	6.91	0.001
			SAT > ABS			
1	White matter near the cingulate gyrus	L	−30, −9, 36	22	5.15	0.039
2	Superior frontal gyrus	R	27, 57, 27	21	4.87	0.045
3	Precentral gyrus	L	−21, 24, 48	38	5.29	0.036
4	Inferior parietal lobule	R	30, −42, 51	22	4.79	0.046
5	Supplementary motor area	L/R	−6, −21, 69	28	4.88	0.044

L, left; R, right; MNI, Montreal Neurological Institute.
